# Exploring sources of variability in adherence to guidelines across hospitals in low-income settings: a multi-level analysis of a cross-sectional survey of 22 hospitals

**DOI:** 10.1186/s13012-015-0245-x

**Published:** 2015-04-28

**Authors:** David Gathara, Mike English, Michael Boele van Hensbroek, Jim Todd, Elizabeth Allen

**Affiliations:** KEMRI Wellcome Trust Research Programme, 43640 – 00100 Nairobi, Kenya; Nuffield Department of Medicine, University of Oxford, Oxford, UK; Department of Global Health, Academic Medical Centre, University of Amsterdam, Amsterdam, UK; Department of Population Health, London School of Hygiene and Tropical Medicine, London, UK; Department of Medical Statistics, London School of Hygiene and Tropical Medicine, London, UK

**Keywords:** Intra-class correlation, Variability, Paediatrics, Hospitals, Clinicians, Low-income settings, Multi-level models, Pneumonia, Malaria, Diarrhoea/dehydration

## Abstract

**Background:**

Variability in processes of care and outcomes has been reported widely in high-income settings (at geographic, hospital, physician group and individual physician levels); however, such variability and the factors driving it are rarely examined in low-income settings.

**Methods:**

Using data from a cross-sectional survey undertaken in 22 hospitals (60 case records from each hospital) across Kenya that aimed at evaluating the quality of routine hospital services, we sought to explore variability in four binary inpatient paediatric process indicators. These included three prescribing tasks and use of one diagnostic. To examine for sources of variability, we examined intra-class correlation coefficients (ICC) and their changes using multi-level mixed models with random intercepts for hospital and clinician levels and adjusting for patient and clinician level covariates.

**Results:**

Levels of performance varied substantially across indicators and hospitals. The absolute values for ICCs also varied markedly ranging from a maximum of 0.48 to a minimum of 0.09 across the models for HIV testing and prescription of zinc, respectively. More variation was attributable at the hospital level than clinician level after allowing for nesting of clinicians within hospitals for prescription of quinine loading dose for malaria (ICC = 0.30), prescription of zinc for diarrhoea patients (ICC = 0.11) and HIV testing for all children (ICC = 0.43). However, for prescription of correct dose of crystalline penicillin, more of the variability was explained by the clinician level (ICC = 0.21). Adjusting for clinician and patient level covariates only altered, marginally, the ICCs observed in models for the zinc prescription indicator.

**Conclusions:**

Performance varied greatly across place and indicator. The variability that could be explained suggests interventions to improve performance might be best targeted at hospital level factors for three indicators and clinician factors for one. Our data suggest that better understanding of performance and sources of variation might help tailor improvement interventions although further data across a larger set of indicators and sites would help substantiate these findings.

## Introduction

Health systems are making efforts to control variation in care quality to raise overall standards and reduce geographic inequalities [1,2]. To achieve this, one must first be able to evaluate quality at scale and then understand the causes of variation so that these can be rectified. In resource limited settings, there are few data on quality of care but these suggest that quality of care varies greatly across place [3,4]. These wide variations and the factors driving them are, however, rarely examined in low-income settings. In higher income settings, variation in care has been associated with geographic regions or communities [5,6], hospitals or primary care units [7] and physicians [1]. However, few studies explore variability across more than one level [8,9] and most examine variability in mortality which while objective, may not be a good indicator of quality of care provided during any immediate pre-terminal events [10-12]. To examine quality of services, it may be more pertinent to examine indicators of the process of care for variability.

The aim of this work is to explore the degree to which variability in performance of important, recommended practices (indicators of process of care) is associated with the organisational units (hospitals) providing care or potentially attributable to variation between individual clinicians. We use data from Kenya where there have been efforts to improve paediatric hospital care through the development and distribution of evidence-based clinical guidelines for some years [13]. Although these efforts have resulted in some overall improvements, [14] considerable variability still remains [4]. Understanding such variation may help inform future interventions to promote improved care at scale.

## Methods

### Context

The availability and adoption of multi-level modelling (MLM) techniques in health care have made it possible to explore and attribute variation at different levels of aggregation of healthcare data. Multi-level models allow for components of variance analysis and estimation of intra-class correlation coefficients (ICC). The ICC may be used as a measure of total variation in a performance measure in a patient population that can be apportioned to one or more aggregated levels in a model. High ICCs suggest a strong within group correlation of individual observations and large group effects, while small ICCs suggest that observations are similar to independent observations (suggesting no group or clustering effect) [15]. From a health service improvement perspective, therefore, a high ICC suggesting considerable variability in performance associated with a level of aggregation may make the level a key target for improvement efforts when considered together with the absolute level of performance [16]. In this work, we are using ICCs for the purposes of exploring sources of variation in care in Kenyan hospitals recognising that many interventions aiming to promote adoption of new practices, particularly education and training, focus on individual health workers.

#### Survey sites, data collection and sample size

Data used were from a cross-sectional survey of 22 ‘internship training centres’ (hereafter referred to as hospitals) purposively identified by the Ministry of Health from a total population of 40 ‘internship training centres’ seeking an administratively and geographically representative sample across Kenya. In brief, the aim of the parent study was to undertake a comprehensive assessment that compared the current practices in internship training hospitals with evidence-based guidelines with an intention of identifying priority areas in need of improvement and to provide recommendations on strategies to improve care. The parent study (described in full elsewhere, [4,17]) aimed at retrieving 60 paediatric inpatient case records per hospital for retrospective chart review. This would allow reporting 50% to 10% correct performance across hospitals with a precision of ±7.5%, adjusted for clustering within hospitals, with a minimum of 12 to 4 cases, respectively, for predefined disease-specific indicators. This approach also meant that the distribution of cases with different diagnoses (case mix) varied across hospitals due to their epidemiological diversity (see Table 1). Each patient record was linked to a specific hospital code and assigned a unique, anonymous clinician code linking patients seen by specific clinicians within each hospital. This clinician code was linked to a separately collected database containing clinician characteristics (age group, cadre-clinical officer, medical officer/paediatrician, work duration and gender). The available case records per hospital, total clinicians and average patients per clinician for each of the disease-related indicators of interest are presented in Table 1.

**Table 1:** **Distribution of cases across indicators**

	**Outcome 1**	**Outcome 2**	**Outcome 3**	**Outcome 4**
	**Prescription of quinine loading dose for malaria**	**Dosing accuracy of crystalline penicillin for pneumonia**	**Prescription of zinc for diarrhoea or dehydration**	**HIV testing for all admitted patients**
Total case available	433	597	271	1298
Total cases with clinician data (%)	368 (85%)	468 (78%)	206 (76%)	1036 (80%)
Hospitals (No.)	19^a^	22	22	22
Mean *n*° of cases per hospital	20	27	12	59
Range of cases per hospital	1–51	13–39	4–27	54–61
Clinicians (No.)	187	226	153	337
Proportion of clinicians with one case	27%	19%	16%	34%
Mean *n*° of clinicians across hospital	8.5	10	7	15
Range of clinicians across hospital	1–26	3–21	3–11	5–30
Range of cases per clinician	1–11	1–20	1–10	1–46

^a^Three sites in non-malaria endemic areas had no cases.

#### Process indicators

We identified process indicators linked to common and important diagnoses in Kenyan hospitals [18,19] representing tasks that are expected functions of the admitting clinician and for which there are clear standards for clinical compliance. Specifically, we sought to explore the variability in three prescribing indicators that included the following: i) prescription of a quinine loading dose for children with malaria; ii) prescription of correct dose per kilogram body weight of crystalline penicillin for children with pneumonia; iii) prescription of zinc for children with diarrhoea/dehydration; and separately, one diagnostic indicator, HIV testing for all children admitted to hospital as is required by national policy. Confirmation of the availability of these drugs or diagnostic tests in the hospitals studied at the time of survey has previously been reported [4].

#### Covariate definitions

Age was categorised into 2–11 and 12–59 months as most guideline recommendations use these age cut-offs. The number of diagnoses made at admission (co-morbidities) was categorised into no co-morbidity (4% (52)), one co-morbidity (49% (635)), two co-morbidities (37% (477)) and three to five co-morbidities (10% (134)). For clinician characteristics, cadre was collapsed into the main cadres in hospitals, clinical officers (62%; 180/291) and medical officers (38%; 181/291 which included 3 clinicians with specialised paediatric training). Similarly, only 16% (46/290) of the clinicians had 2 or more years’ experience; therefore, experience was coded as a binary variable representing internship (0–1 year, 244 (84%)) and post internship (2 or more years, 46 (16%)).

#### Analysis

We initially present overall proportions across hospitals and accompanying 95% confidence intervals (CI) adjusted for clustering at the hospital level for each of the four indicators to illustrate aggregate performance and variability across sites. Subsequently, multi-level mixed models are fitted to explore whether the variability in performance observed is primarily driven by hospital level factors and differences in performance between clinicians in each hospital or is associated with patient level factors. For this, we used a nested model of patients within clinicians within hospitals.

For each of the indicators, four models were specified. The first (model 1) was a two-level model of patients within hospitals with no covariates. The second model (model 2) was a three-level model of patients nested within clinicians nested within hospitals with no covariates that aimed to demonstrate the overall variability at hospital and clinician levels combined. In the third model (model 3), we introduced patient level covariates to model 2 as fixed effects in three separate steps to explore the effect of case mix on the variability observed: a) Step 1 - Age and gender were added because they are not influenced by either hospital or clinician behaviour, b) Step 2 - Disease severity and co-morbidity were added because these may vary by hospital and their presence may influence clinician behaviour, c) Step 3 - All patient level covariates, disease severity, co-morbidity, age and gender, were added to explore the overall effect of patient level covariates. However, for HIV testing which is a diagnostic indicator, disease severity was not included. Finally, in the last model (model 4), we explored the impact of clinician characteristics (gender, age and experience) by adding these as fixed effects to model 2 (first, separately and then all together). Likelihood ratio tests (LRT) were used to compare models 3 and 4 against model 2 to explore whether adding any of these fixed effects improved overall model fit. We also examined the magnitude of change in the ICCs to try and understand how levels and covariates contributed to the variability explained by the models. We present ICC estimates representing total variability explained by the model; therefore, changes in ICC estimates observed after adding the clinician level demonstrate the additional variability explained by the clinician level after allowing nesting of clinicians within hospitals (the difference in ICC estimated in the models with (model 2) and without (model 1) clinicians). In the same way, we can contrast the ICCs from models 3 and 4 with model 2 when fixed effects are introduced. Although we did not have any formal reference point to decide if the ICC had changed to an important degree, we considered absolute changes of more than 25% at a level after including a fixed effect as a change of possible interest. For a subset of cases varying between 24% and 15% across the indicators (see Table 1), it was difficult to link case records with data on the clinician characteristics. Therefore, caution is required when interpreting the comparison of model 2 and model 4 ICC estimates as there were differences in the number of observations.

The *XTMELOGIT* procedure in Stata version 13 for binary outcomes was used for multi-level modelling. The ICCs were calculated using the latent variable method supported by Snijders and Bosker that converts the level 1 variance from the probability scale to the logistic scale on which level 2 (clinician) and level 3 (hospital) are expressed. The standard logistic distribution has a variance of π^2^/3 = 3.29; hence, this can be taken as the level 1 variance. Since levels 1, 2 and 3 variances are on the logistic scale, the following formula was used to estimate ICC at different levels:

$$ \mathrm{I}\mathrm{C}{\mathrm{C}}_{\mathrm{hospital}} = \mathrm{varianc}{\mathrm{e}}_{\mathrm{hospital}}/\left(\mathrm{varianc}{\mathrm{e}}_{\mathrm{hospital}} + \mathrm{varianc}{\mathrm{e}}_{\mathrm{clinician}} + 3.29\right) $$

$$ \mathrm{I}\mathrm{C}{\mathrm{C}}_{\mathrm{clinician}\ \mathrm{and}\ \mathrm{hospital}} = \left(\mathrm{varianc}{\mathrm{e}}_{\mathrm{clinician}} + \mathrm{varianc}{\mathrm{e}}_{\mathrm{hospital}}\right)/\left(\mathrm{varianc}{\mathrm{e}}_{\mathrm{hospital}} + \mathrm{varianc}{\mathrm{e}}_{\mathrm{clinician}} + 3.29\right). $$

To provide plausible ranges of ICC estimates around each estimate, 95% confidence intervals (CI) around the ICCs were constructed using a normal approximation of the logit transformation of the ICC estimates.

## Results

The overall performance for indicators pooled across hospitals is reported in Table 2 and was above 65% for the prescription indicators but poor for HIV testing at 12%. Performance at hospital level varied greatly across all four indicators (although small sample sizes were observed at hospital level for quinine and zinc indicators), for instance, zinc prescription ranged between 29% and 92% amid the best and worst hospitals, while HIV testing for all admitted children ranged from 0% to 47%.

**Table 2:** **Performance of the various outcomes pooled across hospitals**

**Indicator**	**Cases available**	***n*** **(% [95% CI])**	**Median (IQR) for hospital specific proportions**	**Range hospital specific estimates**
Quinine loading dose for malaria patients*	433	320 (74 [62–83])	72 [30–86]	0 to 100
Zinc prescription for diarrhoea/dehydration	271	179 (66 [57–74])	62 [53–80]	29 to 92
Correct crystalline penicillin dose for pneumonia	597	395 (92 [88–95])	95 [90–100]	77 to 100
HIV testing for all admitted children	1298	156 (12 [7-19])	8 [2-16]	0 to 47

^a^From 19 out of the 22 hospitals.

We observed quite different patterns across the indicators for the proportion of variability explained by the different levels in the models. We thus discuss the variability observed for each indicator across the different levels separately with the detailed results on the ICC estimates across the different levels and indicators being presented in Table 3. For models 3 and 4, only models including all covariates at patient and clinician levels will be presented because the full model with all covariates was at least as good a fit as partial models with no appreciable difference in ICC results.

**Table 3:** **Intra-class correlation coefficients for total variability explained by the model for various levels and covariate adjustments across indicators**

**Model**	**Model specific ICC estimate (95% CI)**
Prescription of quinine loading dose for malaria patients	
Two level models with no covariates (model 1)	
Hospital level (ignoring clinician)	0.30 (0.15–0.45)
Nested model with no covariates (model 2)	
Nested model of clinicians within hospitals	0.40 (0.20–0.64)
Nested model with fixed effects for patient characteristics (model 3)	
Age, gender, severity and co-morbidity	0.43 (0.22–0.66)
Nested model with fixed effects for clinician characteristics (model 4)	
Gender, experience and cadre^a^	0.36 (0.15–0.65)
Prescription of correct dose of crystalline penicillin for pneumonia patients	
Two level models with no covariates	
Hospital level (ignoring clinician)	0.07 (0.01–0.44)
Nested model with no covariates	
Nested model of clinicians within hospitals	0.26 (0.05–0.68)
Nested model with fixed effects for patient characteristics	
Age, gender, severity and co-morbidity	0.23 (0.04–0.68)
Nested model with fixed effects for clinician characteristics	
Gender, experience and cadre^a^	0.26 (0.05–0.72)
Prescription of zinc for diarrhoea patients	
Two level models with no covariates	
Hospital level (ignoring clinician)	0.09 (0.03–0.27)
Nested model with no covariates	
Nested model of clinicians within hospitals	0.11 (0.02–0.46)
Nested model with fixed effects for patient characteristics	
Age, gender, severity and co-morbidity	0.14 (0.01–0.63)
Nested model with fixed effects for clinician characteristics	
Gender, experience and cadre^a^	0.10 (0.03–0.33)
HIV testing for all children admitted	
Two level models with no covariates	
Hospital level (ignoring clinician)	0.43 (0.24–0.64)
Nested model with no covariates	
Nested model of clinicians within hospitals	0.43 (0.24–0.64)
Nested model with fixed effects for patient characteristics	
Age, gender and co-morbidity	0.42 (0.24–0.63)
Nested model with fixed effects for clinician characteristics	
Gender, experience and cadre^a^	0.48 (0.27–0.70)

^a^Model 4 has fewer observations due to missing clinician characteristic data.

In the models for prescription of quinine loading dose, the majority of the variability that can be explained by the model is at the hospital level. However, the total variability explained after allowing for nesting of clinicians within hospitals (model 2, ICC estimate of 0.40) increased compared with that of model 1 (ICC estimate of 0.30). This suggests the clinician level also explains a sizeable amount of the total variability observed. Adjusting for patient level and clinician level covariates did not alter this interpretation on the sources of variability.

Comparing ICC estimates from model 1 (ICC = 0.07) and model 2 (ICC = 0.26) for prescription of correct dose of crystalline penicillin, more of the total variability observed could be attributed to the clinician level. Inclusion of patient level and clinician level covariates again did not result in substantial changes in the ICC estimates.

For prescription of zinc for diarrhoea patients, the variability explained by the models was generally low with more of the variability observed attributable to the hospital level. Adjusting for patient level covariates resulted in 25% change in ICC (from 0.11 to 0.14) suggesting that patient level covariates may help explain variability better, and the ICC increased through reduction of the residual variability in the model. However, there was no evidence that adjusting for clinician level covariates explained variability better.

All of the variability observed for HIV testing of children admitted was at the hospital level. Further, there was no evidence that adjusting for patient level and clinician level covariates explained variability better.

Overall, the effect of patient level covariates on the proportion of variability associated with a level varied across indicators. However, across all outcomes, there were no absolute changes in ICCs of greater than 25% after adjusting for patient level covariates except for zinc prescription. Similarly, after adjusting for clinician level covariates, only marginal changes in ICCs were observed. Although there were differences in the ICC estimates between model 2 and model 4, these differences may have arisen due to varying numbers of observations between these two models. However, restricting analyses of both models to complete cases provided similar results (data not shown).

## Discussion

The primary purpose of this analysis was to explore variability in performance of important recommended clinical practices, captured as process indicators that may be associated with organisational units (hospitals), individual clinicians, or with individual patients. We also explored whether this variation in performance was consistent across different process indicators. The absolute values for ICC varied markedly ranging from 0.48 to 0.08 across the models developed for HIV testing and prescription of zinc, respectively. For prescription of quinine loading dose, prescription of zinc and HIV testing, the hospital level was associated with most of the variability that could be explained by models even after allowing nesting of clinicians within hospitals. However, for prescription of quinine, an appreciable amount of variability was explained by the clinician level, while for the prescription of crystalline penicillin, most of the variability explained was at the clinician level.

What are the causes of the different patterns of observed variation in our study? For HIV testing where the government policy is that all children admitted to hospital should be tested for HIV, there was no evidence of any variability at the clinician level. Anecdotal evidence and prior work [13] suggest that supportive supervision and commitment of the hospital leadership to enforce such policies are important in adoption since testing kits are rarely missing in hospitals and were available at the time of study [4]. However, it is also clear that where performance is zero in a facility (as it was in five hospitals for this indicator), there can be no clinician dependent variability. Arguably, for common recommended prescription tasks such as for Zinc and quinine loading dose, differences in local leadership and supervision may explain the predominance of hospital level effects explaining variability. Conversely, more of the observed variability for prescription of correct dose of crystalline penicillin was at the clinician level, and the potential explanation is that the accuracy of dosing captured by this indicator perhaps reflects a task that requires a greater cognitive effort from the clinician in paediatrics as it is a weight-based calculation.

Planning interventions need to be informed by the absolute levels of performance but, we argue, may also be informed by understanding of the sources of variability. The heterogeneity in the sources of variability across indicators suggests potential areas or levels to target for intervention or quality improvement initiatives. Considerable variability explained at hospital level may suggest that interventions aimed at whole organisations are needed, while variability explained at clinician level nested within hospitals may suggest that targeting both hospitals and individual clinicians is required. For instance, it would not seem sensible to rely on clinician training to improve the uptake of HIV testing, particularly training of the type often used in low-income settings of calling individual clinicians to attend centralised, knowledge-focused courses. Thus, perhaps, these data help explain why educational interventions often have modest impacts [20,21]. As a consequence, our findings suggest we should consider more often the complexity of changing practice [13] and interventions that change the way teams and organisations work acknowledging the different factors that explain behaviour [22].

Most ICC estimates from low-income settings are from clinical trials [5,6] and community [5] settings. In the present study, we aimed to describe the distribution of ICCs in routine care using observational data and to evaluate factors that influence the magnitude of ICCs. The estimates we report are relatively high compared to those reported at hospital level in high-income settings (typical ICC < 0.05 in a review by Adams and colleagues [23]). However, they are more consistent with ICC estimates reported across process indicators in low-income settings by Taljaard (median ICC = 0.09) and Haddad (median 0.16 (interquartile range (IQR) 0.07–0.32)) [24,25]. There were no notable difference in ICCs after adjusting for case mix in our study in contrast with the existing literature [16,26], although this largely deals with studies on chronic illnesses with clinically heterogeneous populations [16,27,28].

### Strengths

Our study provides ICC estimates for the acute illness episodes we examined at hospital level that are often lacking for low-income settings. Availability of these estimates should help inform sample size and power calculations for appropriate study designs addressing a recognised challenge of extrapolating ICC estimates to different contexts [29,23]. Secondly, our sample of 22 hospitals is arguably large compared to other studies on quality of care assessment in low-income settings. Finally, by demonstrating the sources of variation, this study highlights the need to understand practice variation in order to target interventions better.

### Limitations

The data we report needs to be interpreted in light of the following limitations. Firstly, this is exploratory work based on a relatively small number of sites, observations and indicators. Paterson and Goldstein suggest at least 25 observations from 25 clusters [30], while Donner and Klar recommend at least 40 clusters [31] for meaningful interpretation. Our estimates from 22 clusters therefore need to be interpreted with caution, and there are further challenges when attempting to estimate variability at the clinician level as 16% to 34% of the clinicians contributed just one observation per indicator. Similar challenges in reliably estimating variability have been reported by Fung [32] and Huang [33]. We also introduced hospitals as a random term although hospitals were not from a random sample. However, we tested the validity of this approach by undertaking the Hausman specification test [34,35] that provided evidence to support this approach across all outcomes.

## Conclusion

Care varies greatly across places with considerable heterogeneity in performance across indicators. More of the variability observed could be explained by hospital than clinician levels, suggesting that interventions aimed at whole organisations may often be more useful than those directed at individual clinicians such as training, although variability amongst individual clinicians may be important to performance in some areas. This type of multi-level analysis may therefore prove useful for recognising sources of variability and suggesting how to target interventions. However, more data across a larger set of indicators and sites are required to better understand variability and substantiate our findings.

## References

[1] Laws RA, Jayasinghe UW, Harris MF, Williams AM, Powell Davies G, Kemp LA (2009). Explaining the variation in the management of lifestyle risk factors in primary health care: a multilevel cross sectional study. BMC Public Health.

[2] Jarman B, Gault S, Alves B, Hider A, Dolan S (1999). Explaining differences in English hospital death rates using routinely collected data. BMJ.

[3] Ayieko P, Okiro EA, Edwards T, Nyamai R, English M (2012). Variations in mortality in children admitted with pneumonia to Kenyan hospitals. PLoS One.

[4] Gathara D, Nyamai R, Were F, Mogoa W, Karumbi J, Kihuba E, et al. Moving towards routine QoC assessment in paediatrics. PLoS One. 2015;10:3,e011117048. 10.1371/journal.pone.0117048 PMC4378956 25822492

[5] Janjua NZ, Khan MI, Clemens JD (2006). Estimates of intraclass correlation coefficient and design effect for surveys and cluster randomized trials on injection use in Pakistan and developing countries. Trop Med Int Health.

[6] Pagel C, Prost A, Lewycka S, Das S, Colbourn T, Mahapatra R (2011). Intracluster correlation coefficients and coefficients of variation for perinatal outcomes from five cluster-randomised controlled trials in low and middle-income countries: results and methodological implications. Trials.

[7] Davis P, Gribben B, Lay-Yee R, Scott A (2002). How much variation in clinical activity is there between general practitioners? A multi-level analysis of decision-making in primary care. J Health Serv Res Policy.

[8] Turenne MN, Hirth RA, Pan Q, Wolfe RA, Messana JM, Wheeler JRC (2008). Using knowledge of multiple levels of variation in care to target performance incentives to providers. Med Care.

[9] Hollingsworth JM, Krein SL, Dunn RL, Wolf JS, Hollenbeck BK (2008). Understanding variation in the adoption of a new technology in surgery. Med Care.

[10] Mohammed MA, Deeks JJ, Girling A, Rudge G, Carmalt M, Stevens AJ (2009). Evidence of methodological bias in hospital standardised mortality ratios: retrospective database study of English hospitals. BMJ.

[11] Lilford RJ, Brown CA, Nicholl J. Use of process measures to monitor the quality of clinical practice. BMJ. 2007,335:648 (September). 10.1136/bmj.39317.641296.AD PMC1995522 17901516

[12] Lilford R, Pronovost P (2010). Using hospital mortality rates to judge hospital performance: a bad idea that just won’t go away. BMJ.

[13] English M, Nzinga J, Mbindyo P, Ayieko P, Irimu G, Mbaabu L (2011). Explaining the effects of a multifaceted intervention to improve inpatient care in rural Kenyan hospitals–interpretation based on retrospective examination of data from participant observation, quantitative and qualitative studies. Implement Sci.

[14] English M, Gathara D, Mwinga S, Ayieko P, Opondo C, Aluvaala J (2014). Adoption of recommended practices and basic technologies in a low-income setting. Arch Dis Child.

[15] Killip S, Mahfoud Z, Pearce K (2004). What is an intracluster correlation coefficient? Crucial concepts for primary care researchers. Ann Fam Med.

[16] Selby JV, Schmittdiel JA, Lee J, Fung V, Thomas S, Smider N (2010). Meaningful variation in performance: what does variation in quality tell us about improving quality?. Med Care.

[17] Aluvaala J, Nyamai R, Were F, Wasunna A, Kosgei R, Karumbi J, et al. Assessment of neonatal care in clinical training facilities in Kenya. Arch Dis Child. 2014;306–423. 10.1136/archdischild-2014-306423 PMC4283661 25138104

[18] Ayieko P, Ntoburi S, Wagai J, Opondo C, Opiyo N, Migiro S (2011). A multifaceted intervention to implement guidelines and improve admission paediatric care in Kenyan district hospitals: a cluster randomised trial. PLoS Med.

[19] Ntoburi S, Hutchings A, Sanderson C, Carpenter J, Weber M, English M (2010). Development of paediatric quality of inpatient care indicators for low-income countries - a Delphi study. BMC Pediatr.

[20] Flodgren G, Conterno LO, Mayhew A, Omar O, Pereira CR, Shepperd S (2013). Interventions to improve professional adherence to guidelines for prevention of device-related infections. Cochrane Database Syst Rev.

[21] Giguère A, Légaré F, Grimshaw J, Turcotte S, Fiander M, Grudniewicz A (2012). Printed educational materials: effects on professional practice and healthcare outcomes. Cochrane Database Syst Rev.

[22] Michie S, van Stralen MM, West R (2011). The behaviour change wheel: a new method for characterising and designing behaviour change interventions. Implement Sci.

[23] Adams G, Gulliford MC, Ukoumunne OC, Eldridge S, Chinn S, Campbell MJ (2004). Patterns of intra-cluster correlation from primary care research to inform study design and analysis. J Clin Epidemiol.

[24] Taljaard M, Donner A, Villar J, Wojdyla D, Velazco A, Bataglia V (2008). Intracluster correlation coefficients from the 2005 WHO Global Survey on Maternal and Perinatal Health: implications for implementation research. Paediatr Perinat Epidemiol.

[25] Haddad SM, Sousa MH, Cecatti JG, Parpinelli MA, Costa ML, Souza JP (2012). Intraclass correlation coefficients in the Brazilian Network for Surveillance of Severe Maternal Morbidity study. BMC Pregnancy Childbirth.

[26] Sullivan CO, Omar RZ, Forrest CB, Majeed A (2004). Adjusting for case mix and social class in examining variation in home visits between practices. Fam Pract.

[27] Ohlsson H, Merlo J (2007). Understanding the effects of a decentralized budget on physicians’ compliance with guidelines for statin prescription–a multilevel methodological approach. BMC Health Serv Res.

[28] Krein SL, Hofer TP, Kerr EA, Hayward RA (2002). Whom should we profile? Examining diabetes care practice variation among primary care providers, provider groups, and health care facilities. Health Serv Res.

[29] Resnicow K, Zhang N, Vaughan RD, Reddy SP, James S, Murray DM (2010). When intraclass correlation coefficients go awry: a case study from a school-based smoking prevention study in South Africa. Am J Public Health.

[30] Paterson L, Goldstein H (1991). New statistical methods for analysing social structures: an introduction to multilevel models. Br Educ Res J.

[31] Donner A, Klar N (2004). Pitfalls of and controversies in cluster randomization trials. Am J Public Health.

[32] Fung V, Schmittdiel JA, Fireman B, Meer A, Thomas S, Smider N (2010). Meaningful variation in performance: a systematic literature review. Med Care.

[33] Huang I-C, Diette GB, Dominici F, Frangakis C, Wu AW (2005). Variations of physician group profiling indicators for asthma care. Am J Manag Care.

[34] Greene WH, William H (2008). Econometric analysis.

[35] Hausman JA (1978). Specification tests in econometrics. ECONOMETRICA.

